# Supplementary material for: Prevalence of obesity and associated risk factors among university students using the newly developed Student Lifestyle and Obesity Risk Questionnaire (SLORQ): a cross-sectional study
Source: PeerJ. 2025 Jun 18;13:e19556. doi: 10.7717/peerj.19556 (PMC12182051; doi:10.7717/peerj.19556)
Supplement: Supplemental Information 1 [file peerj-13-19556-s001.pdf]

| Student Lifestyle and Obesity Risk Questionnaire (SLORQ) | استبيان نمط الحياة ومخاطر السمنة لدي الطلاب (SLORQ) |
|---|---|
| **Section I: Demographic Questions** | **القسم الاول: المعلومات الديموغرافية:** |
| 1. Do you consent to participate in the survey? | 1) هل توافق على المشاركة في الدراسة؟ |
| ○ Yes | ○ نعم |
| ○ No | ○ لا |
| 2. Age group: | 2) الفئة العمرية |
| ○ 18–19-year-old | ○ من 18- 19 سنة |
| ○ 20–21-year-old | ○ من 20- 21 سنة |
| ○ 22–23-year-old | ○ من 22- 23 سنة |
| ○ 24–25-year-old | ○ من 24- 25 سنة |
| ○ 25 and above year old | ○ اكبر من 25 سنة |
| 3. Gender: | 3) النوع |
| ○ Male | ○ رجال |
| ○ Female | ○ نساء |
| 4. Name of the college: (list of colleges at the university) | 4) اسم الكلية |
| ○ College of Applied Medical Sciences | ○ كلية العلوم الطبية التطبيقية |
| ○ College of Medicine | ○ كلية الطب |
| ○ College of Nursing | ○ كلية التمريض |
| ○ College of Education | ○ كلية التربية |
| ○ College of Sciences | ○ كلية العلوم |
| ○ College of Dentistry at Zulfi | ○ كلية طب الاسنان بالزلفي |
| ○ College of Business Administration in Majmaah | ○ كلية إدارة الأعمال بالمجمعة |
| ○ Computer Sciences and Information Technology College | ○ كلية علوم الحاسب وتقنية المعلومات |
| ○ College of Sharia and Law | ○ كلية الشريعة والقانون |
| ○ College of Engineering | ○ كلية الهندسة |
| ○ others | ○ أخري |
| 5. Level: | 5) المستوي الدراسي |
| ○ 1–12 (Choose your current level) | ○ أختار المستوي من (1-12) |
| 6. Region you belong to: | 6) المنطقة الجغرافية التي تنتمي لها |
| ○ Eastern | ○ الشرقية |
| ○ Western | ○ الغربية |
| ○ Southern | ○ الجنوبية |
| ○ Northern | ○ الشمالية |
| ○ Middle | ○ الوسطي |

| | |
|---|---|
| 7. Height (cm): | 7) الطول (بالسنتيميتر) |
| 8. Weight (kg): | 8) الوزن (بالكيلوجرام) |
| 9. BMI: Automatically calculated based on height and weight. | 9) مؤشر كتلة الجسم |
| **Section II: Risk Factors of Obesity Among Students** | **القسم الثاني: عوامل خطر السمنة لدى الطلاب** |
| **Physical Activity and Exercise Domain**<br>Maximum Score:17 | **مجال النشاط البدني والتمرين**<br>**الحد الأقصى للنقاط: 17** |
| 1. What type of physical activities do you engage in? | 1) ما نوع الأنشطة البدنية التي تمارسها؟ |
| ○ Aerobic exercises (e.g., running, swimming) (5) | ○ التمارين الهوائية (مثل االجري والسباحة) (5) |
| ○ Strength training (e.g., weightlifting, resistance bands) (5) | ○ تدريبات القوة (مثل رفع الأثقال و المقاومة) (5) |
| ○ Yoga or Pilates (3) | ○ اليوغا أو البيلاتس (3) |
| ○ Sports (e.g., basketball, soccer) (4) | ○ الرياضة الجماعية (مثل كرة السلة وكرة القدم) (4) |
| ○ Walking only (2) | ○ المشي فقط (2) |
| ○ Housework and shopping (1) | ○ الاعمال المنزلية والتسوق (1) |
| ○ Others _____________ (1) | ○ اخري (1) |
| 2. How often do you engage in physical activity or exercise? | 2) ما هو عدد المرات التي تمارس فيها الرياضة او النشاط البدني في الاسبوع؟ |
| ○ Daily (5) | ○ يوميا (5) |
| ○ 3–4 times per week (4) | ○ من 3-4 مرات في الاسبوع (4) |
| ○ 1–2 times per week (3) | ○ من 1-2 مرة في الاسبوع (3) |
| ○ Rarely (2) | ○ قليل (2) |
| ○ Never (1) | ○ ابدا(1) |
| 3. How hard is your training? | 3) ما شدة الأنشطة البدنية التي تمارسها؟ |
| ○ High intensity (4) | ○ مرتفع الشدة (4) |
| ○ Moderate intensity (3) | ○ معتدل الشدة (3) |
| ○ Low intensity (2) | ○ منخفضة الشدة (2) |
| ○ Sedentary (1) | ○ خمول وكسل (1) |
| 4. Do you participate in regular physical activity on campus? | 4) هل تمارس نشاطًا بدنيًا منتظمًا في الحرم الجامعي؟ |
| ○ Yes (3) | ○ نعم (3) |
| ○ Sometimes (2) | ○ أحيانا (2) |
| ○ No (1) | ○ لا (1) |

| **Dietary Habits Domain**<br>Maximum Score: 20 | **مجال العادات الغذائية**<br>**الحد الأقصى للنقاط: 20** |
|---|---|
| 1. What is your typical daily consumption of fruits and vegetables? | 1) ما هو استهلاكك اليومي النموذجي من الفواكه والخضروات؟ |
| ○ 5 or more servings per day (4) | ○ 5 حصص أو أكثر يوميا (4) |
| ○ 3–4 servings per day (3) | ○ 4-3 حصص يوميا (3) |
| ○ 1–2 servings per day (2) | ○ 2-1 حصص يوميا (2) |
| ○ Less than 1 serving per day (1) | ○ أقل من حصة واحدة في اليوم الواحد (1) |
| 2. What is your primary source of protein? | 2) ما هو مصدرك الأساسي للبروتين؟ |
| ○ Lean meats (e.g., chicken, turkey) (4) | ○ اللحوم الخالية من الدهون (مثل الدجاج والديك الرومي) |
| ○ Fish and seafood (3) | ○ الأسماك والمأكولات البحرية |
| ○ Plant-based sources (e.g., tofu, beans, lentils) (2) | ○ المصادر النباتية (مثل الفول والفاصوليا والعدس) |
| ○ Processed meats (e.g., bacon, sausage) (1) | ○ اللحوم المصنعة (مثل لحم المرتديلا و المقدد والسجق) (1) |
| 3. Do you frequently consume fast food or processed foods? | 3) هل تستهلك بشكل متكرر الوجبات السريعة أو الأطعمة المصنعة؟ |
| ○ Yes, several times per week (1) | ○ نعم، عدة مرات في الأسبوع (1) |
| ○ Occasionally, once a week or less (2) | ○ في بعض الأحيان، مرة واحدة في الأسبوع أو أقل (2) |
| ○ Rarely, only on special occasions (3) | ○ نادراً، فقط في المناسبات الخاصة (3) |
| ○ Never (4) | ○ أبدا (4) |
| 4. Do you eat more sugary foods? | 4) هل تتناول المزيد من الاطعمة السكرية؟ |
| ○ Yes, frequently (1) | ○ نعم، في كثير من الأحيان (1) |
| ○ Occasionally (2) | ○ في بعض الأحيان (2) |
| ○ Rarely (3) | ○ نادرا (3) |
| ○ Never (4) | ○ أبدا (4) |
| 5. Generally, do you feel that you take more calories than you expend? | 5) بشكل عام، هل تشعر أنك تتناول سعرات حرارية أكثر مما تنفقها؟ |
| ○ Yes, often (1) | ○ نعم، في كثير من الأحيان (1) |
| ○ Sometimes (2) | ○ في بعض الأحيان (2) |
| ○ Rarely (3) | ○ نادرا (3) |
| ○ Never (4) | ○ أبدا (4) |
| **Sleep Patterns Domain**<br>Maximum Score: 12 | **مجال أنماط النوم**<br>**الحد الأقصى للنقاط: 12** |
| 1. How many hours of sleep do you typically get each night? | 1) كم عدد ساعات النوم التي تحصل عليها عادة في الليلة الواحدة؟ |

| | |
|---|---|
| ○ Less than 4 hours: (1) | ○ أقل من 4 ساعات في اليوم (1) |
| ○ 4-6 hours: 2 points (2) | ○ من 4-6 ساعات يوميا (2) |
| ○ 6-8 hours: 3 points (3) | ○ من 6-8 ساعات يوميا (3) |
| ○ More than 8 hours: (4) | ○ أكثر من 8 ساعات (4) |
| 2. How often do you feel refreshed and well-rested upon waking up? | 2) كم مرة تشعر بالانتعاش والراحة عند الاستيقاظ؟ |
| ○ always (4) | ○ دائما (4) |
| ○ sometimes (3) | ○ أحيانا (3) |
| ○ Rarely (2) | ○ نادرا (2) |
| ○ Never (1) | ○ أبدا (1) |
| 3. Do you experience difficulty falling or staying asleep? | 3) هل تواجه أي صعوبات في النوم أو الاستمرار فيه؟ |
| ○ Never (4) | ○ أبدا (4) |
| ○ Rarely (3) | ○ نادرا (3) |
| ○ Occasionally (2) | ○ في بعض الاحيان (2) |
| ○ Always (1) | ○ دائما (1) |
| **Metabolic Health Domain**<br>Maximum Score: 3 | **مجال الصحة الأيضية**<br>**الحد الأقصى للنقاط: 3** |
| 1. Have you ever been diagnosed with insulin resistance or prediabetes? | 1) هل سبق أن تم تشخيص إصابتك بمقاومة الأنسولين أو بمقدمات مرض السكري؟ |
| ○ Yes (0) | ○ نعم (0) |
| ○ No (1) | ○ لا (1) |
| 2. Do you have a family history of diabetes or metabolic syndrome? | 2) هل لديك تاريخ عائلي للإصابة بمرض السكري أو متلازمة التمثيل الغذائي؟ |
| ○ Yes (0) | ○ نعم (0) |
| ○ No (1) | ○ لا (1) |
| 3. Do you suffer from any thyroid disorder? | 3) هل تعاني من أي اضطراب في الغدة الدرقية؟ |
| ○ Yes (0) | ○ نعم (0) |
| ○ No (1) | ○ لا (1) |
| **Weight Management Domain**<br>Maximum Score: 11 | **مجال إدارة الوزن**<br>**الحد الأقصى للنقاط: 11** |
| 1. Have you experienced significant weight fluctuations in the past year? | 1) هل واجهت تقلبات كبيرة في الوزن خلال العام الماضي؟ |
| ○ Yes, I have gained weight (1) | ○ نعم، زاد وزني كثيرا (1) |
| ○ Yes, I have lost weight (2) | ○ نعم، نقص وزني كثيرا (2) |
| ○ No, my weight has remained stable (3) | ○ لا، ظل وزني مستقرا (3) |

| | |
|---|---|
| 2. Do you regularly monitor your body weight and waist circumference? | 2) هل تراقب وزن جسمك ومحيط خصرك بانتظام؟ |
| ○ Yes (3) | ○ نعم (3) |
| ○ Sometimes (2) | ○ أحيانا (2) |
| ○ No (1) | ○ لا (1) |
| 3. Have you undergone any weight loss interventions or programs in the past? | 3) هل خضعت لأي تدخلات أو برامج لإنقاص الوزن في الماضي؟ |
| ○ Yes (2) | ○ نعم (2) |
| ○ No (1) | ○ لا (1) |
| 4. Do you have a support system or social network that encourages healthy lifestyle behaviors? | 4) هل لديك نظام دعم أو شبكة اجتماعية تشجع سلوكيات نمط الحياة الصحي؟ |
| ○ Yes (3) | ○ نعم (3) |
| ○ To some extent (2) | ○ الي حد ما (2) |
| ○ No (1) | ○ لا (1) |
| **Environmental and Family Factors Domain** Maximum Score: 12 | **مجال العوامل البيئية والعائلية** **الحد الأقصى للنقاط: 12** |
| 1. Are you aware of the nutritional content of the foods you typically consume? | 1) هل أنت على علم بالمحتوى الغذائي للأطعمة التي تستهلكها عادة؟ |
| ○ Yes (3) | ○ نعم (3) |
| ○ Sometimes (2) | ○ أحيانا (2) |
| ○ No (1) | ○ لا (1) |
| 2. What campus amenities are available within your university? | 2) ما هي مرافق الحرم الجامعي الرياضية المتوفرة داخل جامعتك؟ |
| ○ Gym/Fitness center (4) | ○ صالة الألعاب الرياضية / مركز اللياقة البدنية (4) |
| ○ Sports facilities (3) | ○ المرافق الرياضية (3) |
| ○ Walking/biking paths (2) | ○ مسارات المشي/ ركوب الدراجات (2) |
| ○ Other (1) | ○ أخرى (يرجى التحديد) (1) |
| 3. How is the food availability within your university campus? | 3) كيف يتم توفير الغذاء داخل الحرم الجامعي الخاص بك؟ |
| ○ Healthy options readily available (3) | ○ الخيارات الصحية متاحة بسهولة (3) |
| ○ Limited healthy options, mostly fast food (2) | ○ خيارات صحية محدودة، ومعظمها من الوجبات السريعة (2) |
| ○ Mostly unhealthy options (1) | ○ خيارات غير صحية في الغالب (1) |
| 4. Do you have any chronic medical conditions? | 4) هل تعاني من أي حالات طبية مزمنة؟ |
| ○ Yes (1) | ○ نعم (1) |
| ○ No (2) | ○ لا (2) |

| 5. Do you smoke | 5) هل تدخن السجائر أو تستخدم أي منتجات التبغ الأخرى؟ |
|---|---|
| ○ Yes (1) | ○ نعم (1) |
| ○ Rarely (2) | ○ نادرا (2) |
| ○ No (3) | ○ لا (3) |
| | ○ |
| **Total Scoring** | **مجموع النقاط** |
| • Physical Activity Domain: Maximum = 17 | • مجال النشاط البدني: الحد الأقصى = 17 |
| • Dietary Habits Domain: Maximum = 20 | • مجال العادات الغذائية: الحد الأقصى = 20 |
| • Sleep Patterns Domain: Maximum = 12 | • مجال أنماط النوم: الحد الأقصى = 12 |
| • Metabolic Health Domain: Maximum = 3 | • مجال الصحة الأيضية: الحد الأقصى = 3 |
| • Weight Management Domain: Maximum = 11 | • مجال إدارة الوزن: الحد الأقصى = 11 |
| • Environmental and Family Factors Domain: Maximum = 15 | • مجال العوامل البيئية والعائلية: الحد الأقصى = 15 |
| • Total Maximum Score: 78 | • مجموع الحد الأقصي من النقاط: 78 |

| Grading system and interpretation<br>نظام الدرجات والتفسير | | |
|---|---|---|
| For the overall maximum score of 78, the following grading ranges apply:<br>للحصول على الدرجة القصوى الإجمالية البالغة 78، تنطبق نطاقات الدرجات التالية: | | |
| Score Range<br>مدي الدرجات / | Percentage Range<br>مدي النسبة المئوية/ | Interpretation / التفسير |
| 66–78 | 85–100% | Excellent overall health behaviors<br>سلوكيات صحية عامة ممتازة |
| 55–65 | 70–84% | Good health behaviors<br>سلوكيات صحية جيدة |
| 39–54 | 50–69% | Moderate health behaviors<br>سلوكيات صحية معتدلة |
| 24–38 | 30–49% | Poor health behaviors; needs improvement<br>سلوكيات صحية سيئة؛ يحتاج إلى تحسين |
| <24 | <30% | Very poor health behaviors; high risk<br>سلوكيات صحية سيئة للغاية؛ مخاطر عالية |

| Domain/ المجال | Max Score/ الدرجة القصوي | Excellent (85–100%) ممتاز | Good (70–84%) جيد | Fair (50–69%) مقبول | Poor (30–49%) ضعيف | Critical (<30%) ضعيف جدا |
|---|---|---|---|---|---|---|
| Physical Activity/ مجال النشاط البدني | 17 | 15–17 | 12–14 | 9–11 | 5–8 | 0–4 |
| Dietary Habits/ مجال العادات الغذائية | 20 | 17–20 | 14–16 | 10–13 | 6–9 | 0–5 |
| Sleep Patterns/ مجال أنماط النوم | 12 | 10–12 | 8–9 | 6–7 | 4–5 | 0–3 |
| Metabolic Health/ مجال الصحة الأيضية | 3 | 3 | 2 | NG | 1 | 0 |
| Weight Management/ مجال إدارة الوزن | 11 | 9–11 | 8–9 | 6–7 | 4–5 | 0–3 |
| Environmental & Family/ مجال العوامل البيئية والعائلية | 15 | 13–15 | 11–12 | 8–10 | 5–7 | 0–4 |

- NG; not graded / لا درجات
